# Correction: Castro et al. Synthesis, Characterization, and Optimization Studies of Polycaprolactone/Polylactic Acid/Titanium Dioxide Nanoparticle/Orange Essential Oil Membranes for Biomedical Applications. *Polymers* 2023, *15*, 135

**DOI:** 10.3390/polym18030379

**Published:** 2026-01-30

**Authors:** Jorge Ivan Castro, Stiven Astudillo, Jose Herminsul Mina Hernandez, Marcela Saavedra, Paula A. Zapata, Carlos Humberto Valencia-Llano, Manuel N. Chaur, Carlos David Grande-Tovar

**Affiliations:** 1 Grupo de Investigación SIMERQO, Departamento de Química, Universidad del Valle, Calle 13 No. 100-00, Santiago de Cali 76001, Colombia; 2 Grupo de Materiales Compuestos, Escuela de Ingeniería de Materiales, Facultad de Ingeniería, Universidad del Valle, Calle 13 No. 100-00, Santiago de Cali 760032, Colombia; 3 Grupo de Polímeros, Facultad de Química y Biología, Universidad de Santiago de Chile, USACH, Santiago 9170020, Chile; 4 Grupo Biomateriales Dentales, Escuela de Odontología, Universidad del Valle, Calle 4B # 36-00, Cali 76001, Colombia; 5 Grupo de Investigación de Fotoquímica y Fotobiología, Facultad de Ciencias, Universidad del Atlántico, Carrera 30 Número 8-49, Puerto Colombia 081008, Colombia

## Errors in Figures

In the original publication [1], there were two mistakes in Figures 7 and 8 as published.

During the preparation of the original article [1], there was an error in Figure 7. A macroscopic photograph of a biomodel implanted with different scaffolds was unintentionally repeated from our previous work [2].

This mistake arose because, in our research, we routinely employ the same animal biomodels to evaluate multiple materials simultaneously. Specifically, we use a validated subcutaneous pocketing technique that enables the creation of up to ten independent dorsal pockets per animal, each implanted with a distinct material. In some experimental designs, two or three different systems are evaluated in the same biomodel. This methodology enhances resource efficiency, reduces biological variability among animals, and allows comparative evaluation across multiple materials. Importantly, it aligns with the internationally accepted 3R principles (Replacement, Reduction, and Refinement) for ethical animal experimentation.

The figure in question depicts a macroscopic view of the dorsal implantation area. Such images are included only as preliminary visual references of tissue response and healing, serving as complementary context to the histological analyses which remain the primary and valid scientific evidence supporting the study’s conclusions.

The corrected version of Figure 7 is provided below. We confirm that this correction does not alter the validity of the data, the results, or the conclusions of the article. The amendment leaves the core findings and interpretations intact, ensuring that data integrity and scientific outcomes remain unchanged.

Additionally, a histological image was inadvertently duplicated between panels F1B and F4B of Figure 8. This error was unintentional and occurred during the selection and assembly of images for the final figure, due to the large number of images generated for each formulation. Specifically, implants were evaluated at 30, 60, and 90 days, resulting in a total of 209 histological images.

The specific mistake occurred because the image corresponding to panel B of formulation F1 at 30 days was accidentally duplicated in panel B of formulation F4, also at 30 days. Both images were stained using Masson’s trichrome, which explains the strong similarity in coloration and morphological features and contributes to the oversight during the image selection process.

The corrected version of Figure 8 is provided below. It is important to emphasize that this error occurred exclusively during the organization and selection of images. It does not affect data acquisition, histological processing, scientific analysis, or the validity of the results and conclusions presented in the article.

**Figure 8:**
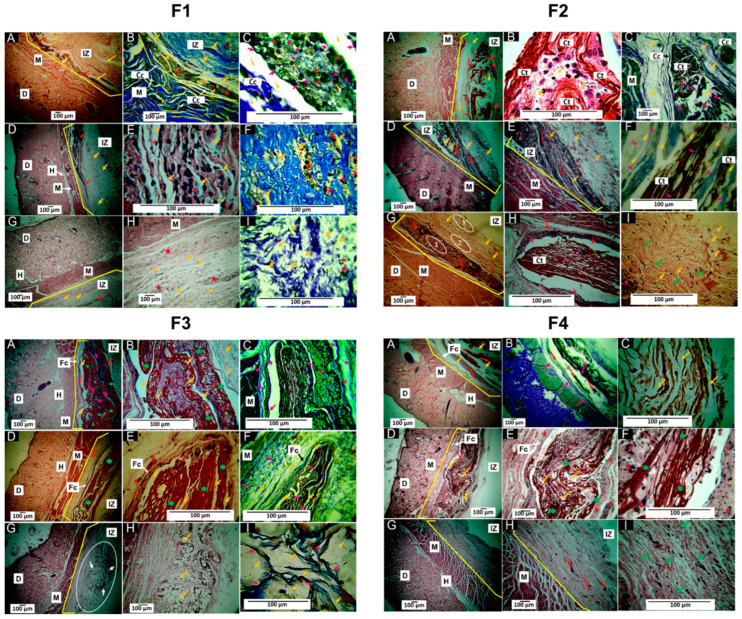
Histological analysis of F1, F2, F3, and F4 membranes. (**A**–**C**): 30-day implantations. (**D**–**F**): 60-day implantations. (**G**–**I**): Implantations at 90 days. Panel F1: (**A**): Image at 4× HE technique. (**B**): Image at 10× MT technique. (**C**): Image at 100× MT technique. (**D**): Image at 4× HE technique. (**E**): Image at 40× HE technique. (**F**): Image at 40× MT technique. (**G**): Image at 4× HE technique. (**H**): Image at 10× HE technique. (**I**): Image at 40× MT. Panel F2: (**A**): Image at 4× HE technique. (**B**): Image at 40× HE technique. (**C**): Image at 10× MT technique. (**D**): Image at 4× HE technique. (**E**): Image at 10× HE technique. (**F**): Image at 40× MT technique. (**G**): Image at 4× HE technique. (**H**): Image at 40× HE technique. (**I**): Image at 40× HE. Panel F3: (**A**): Image at 4× HE technique. (**B**): Image at 40× HE technique. (**C**): Image at 40× MT technique. (**D**): Image at 4× HE technique. (**E**): Image at 40× HE technique. (**F**): Image at 40× MT technique. (**G**): Image at 4× HE technique. (**H**): Image at 10× HE technique. (**I**): Image at 40× MT technique. Panel F4: (**A**): Image at 4× HE technique. (**B**): Image at 4× MT technique. (**C**): Image at 40× HE technique. (**D**): Image at 4× HE technique. (**E**): Image at 10× HE technique. (**F**): Image at 40× HE technique. (**G**): Image at 4× HE technique. (**H**): Image at 10× HE technique. (**I**): Image at 40× HE. D: Dermis. M: Muscle. IZ: Implantation zone. Cc: Connective tissue capsule. Ct: Connective tissue. Fc: Fibrous capsule. H: hypodermis. Red arrow: area with resorbing material. Yellow arrow: Material. Pink arrows: type I collagen fibers. Green arrows: Connective tissue. Red stars: Inflammatory cells. Green stars: Connective tissue. Oval 1: Area with the material in the process of resorption. Oval 2: Zone with less inflammatory activity. Oval 3: Zone with the formation of connective scar tissue. White oval: Histological interest zone. White arrows: material in the process of degradation/resorption. MT: Masson’s trichrome stain. HE: Hematoxylin–Eosin stain.

This correction was approved by the Academic Editor. The original publication has also been updated. 

## Figures and Tables

**Figure 7:**
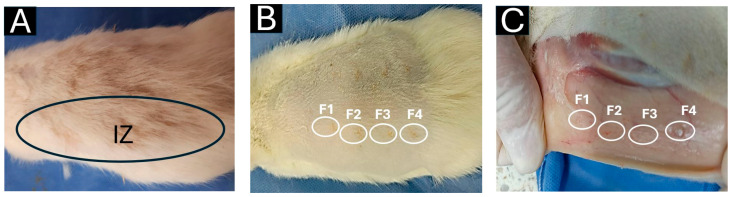
Subdermal dorsal implantation zone. (**A**) Dorsal area with abundant hair. (**B**) Trichotomy of the dorsal region. (**C**) Subdermal implantation area. Black oval: Implantation zone. White circles: Blocks implanted. F1–F4: Formulations 1, 2, 3, and 4. IZ: Implantation zone.
