# Supplementary material for: Synthesis, Characterization, and Optimization Studies of Polycaprolactone/Polylactic Acid/Titanium Dioxide Nanoparticle/Orange Essential Oil Membranes for Biomedical Applications
Source: Polymers (Basel). 2022 Dec 28;15(1):135. doi: 10.3390/polym15010135 (PMC9823686; doi:10.3390/polym15010135)
Supplement: Supplementary file 1 [file polymers-15-00135-s001.zip › polymers-2111195-supplementary.pdf]

polymers

MDPI

Article

Synthesis, Characterization, and Optimization Studies of Polycaprolactone/Polylactic Acid/Titanium Dioxide Nanoparticle/Orange Essential Oil Membranes for Biomedical Applications

Jorge Ivan Castro, Stiven Astudillo, Jose Herminsul Mina Hernandez, Marcela Saavedra, Paula A. Zapata, Carlos Humberto Valencia-Llano, Manuel N. Chaur and Carlos David Grande-Tovar

Table S1. Volatile compounds are expressed as a relative amount in percentage identified in the *Orange essential oil.*

t_{R1}	LRI, DB-5MS		Compound	Relative amount (OEO) (%)
	RI[a]	RI[b]		
**16.1**	**936**	932 [1]	α-Pinene	1.1
**17.9**	975	946[1]	Carphene	1.2
**18.1**	981	974[1]	β-pinene	0.2
**18.5**	990	988[1]	β-myrcene	2.9
**19.1**	1004	1003[2]	Octanal	0.4
**19.5**	1012	1008[1]	Δ^3- careno	0.2
**20.2**	1028	1020[1]	p-cymeno	0.3
**20.5**	1037	1024[1]	limonene	89.6
**21.5**	1062	1054[1]	g-Terpinene	0.1
**23.2**	1101	1095[1]	linalool	0.7
**23.4**	1106	1104[2]	Nornanal	0.1
**24.2**	1126	1119[1]	*trans*-p-Menta-2,8-dien-1-ol	0.1
**24.6**	1138	1134[1]	cis-Limonene epoxide	0.1
**24.8**	1142	1138[2]	limonene trans-epoxide	0.3
**25.2**	1154	1148[1]	Citronellal	0.1
**27**	1200	1189[2]	α-Terpineol	0.2
**27.3**	1207	1201[1]	Decanal	0.7
**27.9**	1223	1217[2]	*Trans*-Carveol	0.1
**28.8**	1250	1246[2]	Carvona	0.3
**30.3**	1291	1286[2]	trans-Anetol	0.2
**30.6**	1299	1298[1]	Carvacrol	0.2
**32.4**	1347	-	$C_{10}H_{18}O_2$	0.2
**34.7**	1410	1409 [2]	Dodecanal	0.2
**37.9**	1501	1496 [1]	Valencene	0.5

[a]Kovats Retention Indices experimental; [b] Kovats Retention Indices from literature.

[1] ADAMS, P. Identification of essential oil components by gas chromatography/mass spectrometry. 4th edición, Allured Publishing Corporation, Carol Stream, Illinois, 2004.

[2] NIST Mass Spectrometry Data Center. http://webbook.nist.gov/chemistry/, available online day month year
